# Cadaveric surgery in core gynaecology training: a feasibility study

**DOI:** 10.1186/s10397-017-1034-0

**Published:** 2018-01-16

**Authors:** Chou Phay Lim, Mark Roberts, Tony Chalhoub, Jason Waugh, Laura Delgaty

**Affiliations:** 1 Obstetrics and Gynaecology, Royal Victoria Infirmary, Newcastle upon Tyne Hospitals NHS Foundation Trust, Newcastle Upon Tyne, NE1 4LP UK; 2 Royal Victoria Infirmary, Newcastle Upon Tyne, UK; 3 Obstetrics and Gynaecology, Northern Deanery, HEE (NE), Newcastle Upon Tyne, UK; 4 Newcastle University, Newcastle Upon Tyne, UK

**Keywords:** Cadaveric surgery, Laparoscopy training, Gynaecological surgery, Surgical confidence

## Abstract

**Background:**

Fresh frozen cadaver training has been proposed as a better model than virtual reality simulators in laparoscopy training. We aimed to explore the relationship between cadaveric surgical training and increased surgical confidence.

To determine feasibility, we devised two 1-day cadaveric surgical training days targeted at trainees in obstetrics and gynaecology. Seven defined surgical skills were covered during the course of the day. The relationship between surgical training and surgical confidence was explored using both quantitative (confidence scores) and qualitative tools (questionnaires).

**Results:**

Participants rated a consistent improvement in their level of confidence after the training. They universally found the experience positive and three overarching themes emerged from the qualitative analysis including self-concept, social persuasion and stability of task.

**Conclusions:**

It is pragmatically feasible to provide procedure-specific cadaveric surgical training alongside supervised clinical training. This small, non-generalisable study suggests that cadaveric training may contribute to an increase in surgical self-confidence and efficacy. This will form the basis of a larger study and needs to be explored in more depth with a larger population.

## Background

It is now an expectation that an integral part of surgical training should include meaningful simulation prior to live patient operating wherever possible. Training time in obstetrics and gynaecology has been reduced over the past 20 years following the introduction of shorter training programmes and work hour restrictions from bodies like the European Working Time Directive (EWTD) and the Accreditation Council for Graduate Medical Education (ACGME). As such there is an increasing opportunity for realistic surgical simulation to improve clinical training [1–9].

There are a number of simulation methods that suit acquisition of various skills. For example, neoprene surgical pads or animal tissue may be used to learn suturing and tissue handling, and laparoscopic ‘boxes’ or virtual simulation may improve hand-eye co-ordination [10]. There is difficulty, however, in simulating core procedures such as salpingectomy, which is one of the mandatory competencies in the curriculum for gynaecology trainees. Animal models lack anatomical accuracy and artificial models lack the realism of live tissue [11]. Fresh frozen cadaver training has been proposed as a better model than virtual reality simulators in laparoscopy training [12].

The use of cadaveric material was at one time a cornerstone of medical training, including undergraduate medical school anatomy teaching. More recently, students and trainees have little, if any, exposure to cadaveric material, those who do often only see prosected or preserved specimens. The concept of ‘procedure-specific’ cadaveric surgical training has been recently introduced in several specialities, including orthopaedics, trauma medicine and head and neck surgery [12–14]. This has become possible by advances in preserving human tissue for teaching purposes. Cadaveric simulation in gynaecology has been applied in some surgical settings such as gynaecological oncology, targeted at advanced surgical trainees and established consultant specialists [7, 8] but not at junior trainees who are still at the stage of familiarising basic surgical skills and procedures.

The benefit of this training intervention is not easy to quantify, but it is postulated that improved self-belief in a task enhances self-confidence [15]. Self-confidence, for trainees, is the trust in one’s abilities, qualities and judgement. Social cognitivist theory refers to this belief of self-ability as ‘perceived self-efficacy’, and this is one of the cognitive mechanisms underlying behavioural change [16]. Importantly, in this context, it is also one of the factors that predict performance success [17]. Literature suggests that those who expect to do well, demonstrating a high belief in their own self-efficacy, are more likely to do well than those who expect to do poorly [18]. Therefore, with the increasing constraints on surgical gynaecological training, educators must identify relevant factors that can increase skill acquisition and self-efficacy, or surgical confidence [16]. In the context of this study, it is anticipated that improved surgical confidence may be linked to surgical expertise and, therefore, competence. For educators, being aware of this relationship and, designing training programmes accordingly, is essential to surgical expertise and, ultimately, patient outcomes [19].

We aimed to explore the relationship between cadaveric surgical training and increased surgical confidence. We report the feasibility of introducing cadaveric surgical training into a basic training programme at year 3. To our knowledge, the use of cadaveric material in basic gynaecology training has never been reported.

## Methods

Using a survey methodology, the relationship between cadaveric surgical training and improved confidence was explored. A mixed methods approach was used, which facilitated the collection of both quantitative and qualitative data.

### Training resources

Postgraduate specialty training in obstetrics and gynaecology within England is run by 13 geographically separate Local Education Training Boards (LETB). Funding to introduce a cadaveric surgery programme for gynaecology trainees in Health Education England North East (HEE NE) was obtained from Health Education England. The programme was run at the Newcastle Surgical Training Centre (NSTC).

The Human Tissue Act 2004 established standards and guidance to institutions carrying out education and training using human cadaveric materials [20]. In collaboration with the department of Anatomy in Newcastle University, the Newcastle Surgical Training Centre (NSTC) is licenced under the Human Tissue Authority (HTA). Details of the philosophy and ethical approval in the use of cadaveric material within this facility were described in another publication [14]. The cadaveric material is stored fresh frozen at − 17 to − 20 °C and defrosted before use.

### Study design

Competency level of obstetrics and gynaecology trainees in the UK follows a standardised 7-year curriculum set out by the Royal College of Obstetricians and Gynaecologists (RCOG) [21, 22]. We devised two 1-day surgical training days targeted at trainees (year 3) to cover the surgical competencies in the training programme of the RCOG [21, 22]. The first day was set to be within the first 3 months of the training year and the second was set to be 8 months later. The content of the days were the same. We exclude any candidates who have taken a significant time away from the training programme within this timeframe for example for parental leave or long-term sick leave to maintain a homogenous level of training experience within the interval time period. Ethical consideration of the study was reviewed and approved from the Newcastle Clinical Research Facility. Informed consent was obtained from all participants included in the study.

The training days ran from 0900 to 1700 h. Each station was supplied with one female cadaver torso (assessed by CT scan to confirm that a uterus was present) allocated to two or three delegates and one trainer. The delegates were encouraged to perform the surgical procedures as the primary surgeon under the supervision and guidance of the trainer at their station. Seven defined surgical skills were covered during the course of the day including tubal clip sterilisation (laparoscopic), laparoscopic salpingectomy, laparoscopic oophorectomy, laparoscopic specimen retrieval, opening and closing the abdomen (suprapubic transverse incision), optimising the surgical field (open and laparoscopic), and abdominal hysterectomy.

The trainers were all consultant gynaecological surgeons from approved training units across the HEE NE region. All the members of faculty attended a pre-course briefing at the NSTC and were all asked to follow a set programme and standard technique for the purpose of this programme to keep training similar across cadaver groups. This is so that delegates are not confused by the variety of surgical techniques and preferences among different consultant trainers.

### Outcome measures

We collected two types of data to investigate confidence. All data was anonymised:

1. Quantitative: self-assessed surgical confidence score (SCS)
2. Qualitative: Evaluation form

### Analysis: surgical confidence score (SCS)

**Fig. 1:**
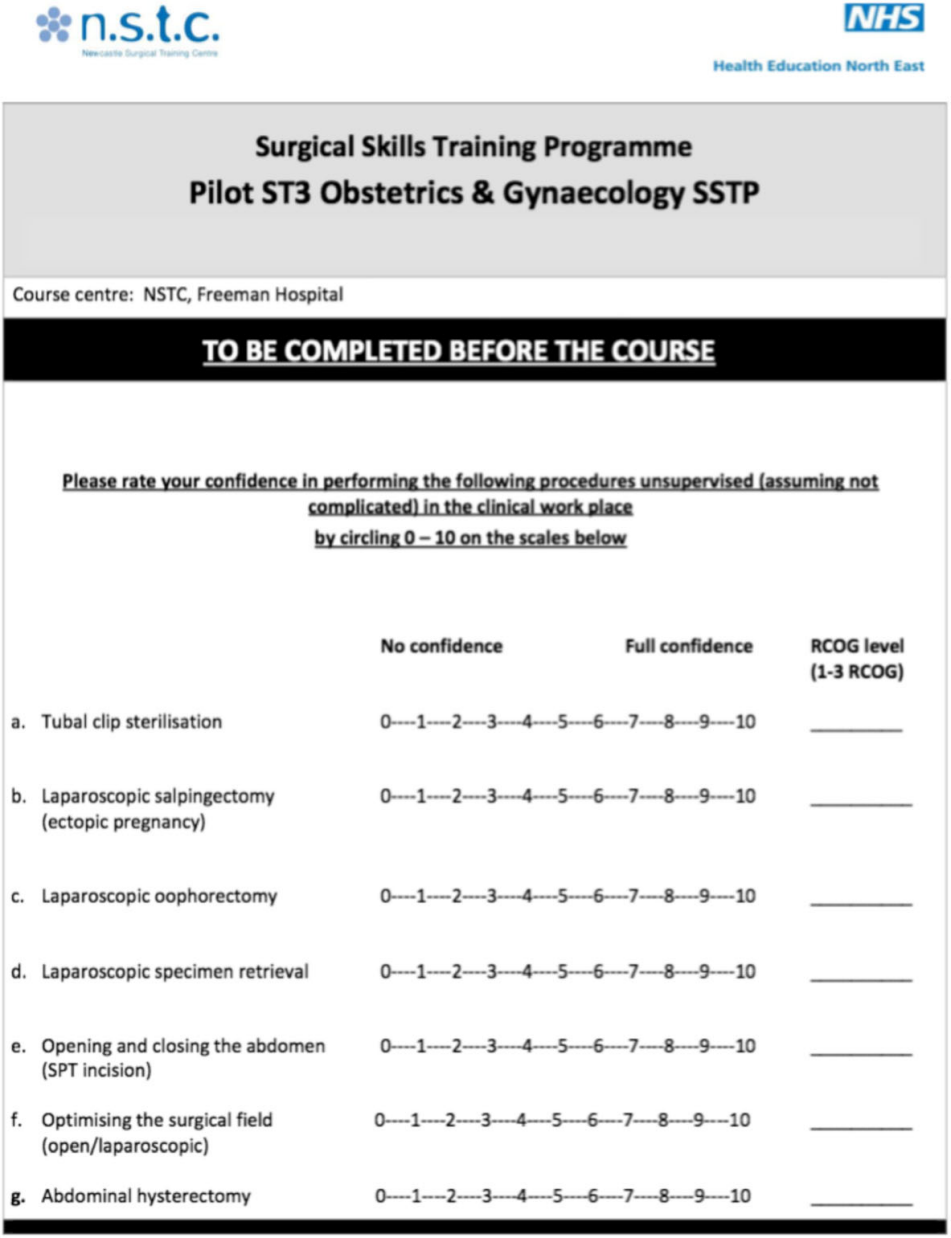
Surgical confidence score (SCS)

We designed a surgical confidence score (SCS) system to quantify the level of confidence the delegates had in approaching surgical cases (Fig. 1). The delegates score their level of confidence on a Likert scale from 0 to 10 (0 meaning ‘no confidence’ and 10 meaning ‘full confidence’) on the SCS. This was done at the beginning and the end of each day of the programme. For each of the surgical skills, we calculated the mean of the SCS and used two-tailed paired Student’s *t* test to determine the change in the delegates’ confidence.

### Analysis: qualitative evaluation form

We invited the delegates to complete an evaluation at the end of each day. We asked the delegates whether they felt that the course delivered value for money, whether they felt the course should be funded from the trainee study leave budget in the future and general questions concerning individual experiences. The evaluation used a combination of simple rating scales for satisfaction and free text questions allowing for the collection of rich data. All free text data was analysed broadly following a method of qualitative content analysis described by Cohen et al. [23]. Codes were generated from the rough data and overarching themes or labels emerged. The themes were reviewed and refined.

## Results

**Fig. 2:**
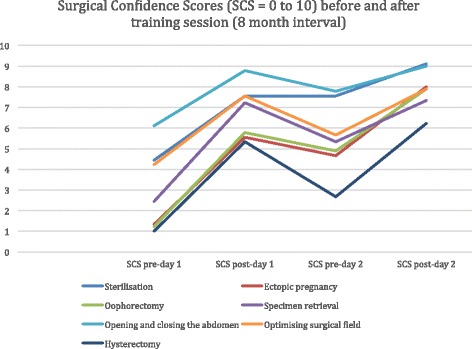
SCS linear graph

Data from the nine trainees at ST year 3 level within the HEE NE training programme who attended both days of the 2-day programme are reported. Figure 2 shows the linear changes in mean delegate SCS for the seven surgical competencies over the four time points. This is presented as a visual representation of all the results. A more detailed analysis is given in Tables 1, 2, 3 and 4 and in the text below.

**Table 1:** Analysis of Day 1 and Day 2 mean SCS

Procedures	Day 1 SCS			Day 2 SCS			Overall change Pre-day 1 to post day 2
Trainees = 9	Pre day 1	Post day 1	Diff day 1	Pre day 2	Post day 2	Diff day 2	
Sterilisation	4.44	7.56	3.12	7.56	9.11	1.55	4.67**
Ectopic	1.33	5.56	4.23	4.67	8.00	3.33	6.67****
Oophorectomy	1.22	5.78	4.56	4.89	7.89	3.00	6.67****
Specimen Retrieval	2.44	7.22	4.78	5.33	7.33	2.00	4.89***
Opening and Closing	6.11	8.78	2.67	7.78	9.00	1.22	2.89*
Optimising surgical field	4.22	7.56	3.34	5.67	7.89	2.22	3.67***
Hysterectomy	1.00	5.33	4.43	2.67	6.22	3.55	5.22***

(* = *p* < 0.05, ** = *p* < 0.01, *** = *p* < 0.001, **** = *p* < 0.0001)

**Table 2:** Sub analysis of SCS Post Day 1 to Pre Day 2

Procedures	Mean SCS after Day 1	Mean SCS before Day 2	Difference in Mean SCS	Student t-test *p* values
Sterilisation	7.56	7.56	0.00	1.000
Ectopic	5.56	4.67	−0.89	0.069
Oophorectomy	5.78	4.89	−0.89	0.138
Specimen Retrieval	7.22	5.33	−1.89	*0.033
Opening and Closing	8.78	7.78	−1.00	0.108
Optimising surgical field	7.56	5.67	−1.89	*0.023
Hysterectomy	5.33	2.67	−2.67	**0.007

(* = *p* < 0.05, ** = *p* < 0.01)

**Table 3:** Sub analysis of SCS Pre-Day 1 and Pre-Day 2

Procedures	Mean SCS before Day 1	Mean SCS before Day 2	Difference in Mean SCS	Student t-test *p* values
Sterilisation	4.44	7.56	3.11	*0.022
Ectopic	1.33	4.67	3.33	*0.022
Oophorectomy	1.22	4.89	3.67	**0.005
Specimen Retrieval	2.44	5.33	2.89	*0.022
Opening and Closing	6.11	7.78	1.67	0.179
Optimising surgical field	4.22	5.67	1.44	0.089
Hysterectomy	1.00	2.67	1.67	*0.028

(* = *p* < 0.05, ** = *p* < 0.01)

**Table 4:** Evaluation forms response

Satisfaction score overall rating	5	4	3	2	1
Cadaveric session 1	8	1	0	0	0
Cadaveric session 2	8	1	0	0	0
	YES		NO		
Do you feel the course should be funded from the study leave budget in the future?	9		0		
Did you find cadaveric surgery training unpleasant?	0		9		

### Surgical confidence scores

There was a significant improvement in the mean SCS in all the seven surgical skills assessed. Table 1 shows the mean SCS for the nine delegates on each of the surgical skills across both training days and the respective improvement in the mean SCS. There was a mean increase in the SCS, ranging from 2.89 (*p* < 0.05) for opening and closing the abdomen to 6.67 (*p* < 0.0001) for ectopic pregnancy, with statistically significant improvements in SCS for all seven surgical skills assessed.

### Sub-analysis of SCS (post-day 1 to pre-day 2)

Table 2 presents the data and statistical analysis that allows comparison of SCS at the end of day 1 to the start of day 2. This was designed to assess whether SCS was retained over the 8-month interval between training days.

Between the end of the first training day and the start of the second training day, there was a general trend of reduction in the SCS.

We were unable to report specific details on each delegates’ case volume in the interim between the two training days because this information was not collected. However, all delegates remained in active training under the nationally approved specialty training programme during this time frame, with regular opportunity in assisting and performing live surgical cases under close supervision in each of their training hospital unit.

### Sub-analysis of SCS (pre-day 1 and pre-day 2)

Table 3 presents the comparison of SCS before both training days. This was intended to assess whether the baseline SCS on day 2 was higher, given that trainees had already undergone day 1 followed by consolidation clinical training in the 8-month interval. There was a significant improvement for sterilisation, oophorectomy, salpingectomy, specimen retrieval, and abdominal hysterectomy. There was no difference in this comparison in opening and closing the abdomen (*p* = 0.18) and optimising the surgical field (*p* = 0.09).

### Study day evaluation

The evaluation attracted universally positive feedback. None of the delegates found the experience unpleasant. All of the delegates responded to confirm that they would be willing to pay for the programme (cost £550) from their set annual training budget. We present the evaluation results from satisfaction scales in Table 4 with further analysis of the qualitative data below.

### Analysis of qualitative data

i) Self-concept

The participants personalised their experiences suggesting that the opportunity presented allowed them to perform these procedures ‘without fear’ in comparison to a live patient and without ‘feeling silly’. Furthermore, performing the procedures in ‘real time’ was clearly valuable and suggested transferability to actual surgical practice.

ii) Social persuasion

A perhaps unanticipated theme was one of social persuasion. The learners consistently suggested the value of the faculty and how it contributed to their overall gain in confidence. Having the support and guidance of these individuals was essential. Also, the opportunity to ‘discuss the techniques’ both with faculty and their peers added to their experience and self-assurance.

iii) Stability of task

This final theme encompassed the very nature of the learning opportunity with cadaveric specimens. Participants overwhelmingly suggested the experience itself, the ‘hands on’ nature of it and the ‘practical exposure’ was valuable. There were specific codes making up this theme demonstrating the actual learning process in this stable environment. These included the specific procedures they were tasked to perform, the ‘opportunity to identity anatomy’ in cadaveric specimens and ‘running through the actual steps’.

## Discussion

This was intended as a feasibility study, and we have had an opportunity to demonstrate from here that cadaveric surgery improves surgical confidence.

Analysis of SCS at different stages of the study gives some insight into the value of cadaveric surgical simulation training. Firstly, it is noted that there was a universal improvement in confidence scores for all skills assessed (Table 1). The greatest improvement appeared to be for oophorectomy, salpingectomy and hysterectomy. These are procedures that year 3 trainees are often only just starting to undertake as supervised clinical cases, which may explain why the baseline SCS prior to day 1 were lower than for other procedures. Whereas for procedures that year 3 trainees were likely to be already experienced in, such as opening and closing the abdomen and optimising the surgical field (skills used in caesarean section), the baseline SCS prior to day 1 was higher and therefore the overall improvement was less as a result.

Table 2 demonstrates that SCS retention over 8 months was variable for different competencies. This retention of confidence is likely to be related to the trainees’ actual surgical experiences over the 8-month interim. For example, trainees were almost as confident at the start of day 2 as the end of day 1 for procedures that were simple and likely to be a common part of supervised clinical training, such as sterilisation and opening and closing the abdomen. The greatest reduction in SCS between the two training days was for hysterectomy. This is likely to be because hysterectomy is more complex to undertake and trainees have less opportunities for supervised clinical training. Table 3 shows that despite some loss of confidence for some of the surgical competencies, trainees were universally more confident at the start of day 2 than day 1. This would suggest that the intervention of cadaveric simulation combined with supervised clinical training is an effective approach to teaching surgical procedures. Simulation training is an important adjunct rather than an alternative to clinical experience [24] but is likely to be of greatest value when simulation is timed to clinical exposure.

Three main themes emerged from the qualitative data: self-concept, social persuasion and stability of task. These are described in detail in the “Results” section. The responses were overwhelmingly positive, suggesting that trainees felt better equipped to engage with supervised clinical training. Not only did trainees highlight the value of real-time surgery and real anatomy but also the interaction with trainers and working in an environment where mistakes can be made before operating on live patients. This would suggest that it is not just the use of cadavers are important but also the structure of the training days.

Our study report results from the first nine participants to undertake both training days as a group. This was limited in part by the regional workforce for the population and also our intention to study a homogenous group of trainees of similar level of experience. All delegates had more than 2 years but less than 3 years equivalent of full-time training within obstetrics and gynaecology. As this was a pilot intended as a feasibility study with a small sample size, we are cautious in claiming generalisability or drawing conclusions surrounding the benefit of cadaveric surgical training. As a feasibility study, the aim was to produce findings that help determine whether this intervention could be recommended for further studies.

This study suggests that more advanced surgical trainees can be trained with a similar model on more complex advance procedures. The process of training a surgeon to a level of higher confidence can also be supplemented with this model. Our group have begun work in implementing a more regular set of training days around similar models with cadaveric work throughout the training programme for the region.

In the absence of clinical training, confidence level varies greatly [25]. Clanton et al. reported in a study involving 150 medical students in basic surgical skills that there is a strong association between confidence and competence [19]. Hutton et al. reported a similar relationship between confidence and competence in chest tube insertion by junior doctors [26].

The values of surgical simulation training have been established in other medical disciplines [10, 11, 27, 28]. Palter et al. described 25 colorectal surgical residents who received ex vivo simulation training and demonstrated improved technical knowledge and performance in the operating room compared to conventional residency training [29]. Ahmed et al. described a series of 81 urology residents having undergone a cadaveric surgical programme. They reported that human cadaveric surgery was the best mode of simulation-based training with improvements in skills that were transferrable to the operating theatre based on evaluation surveys only [30]. This study may be criticised for its heterogeneity because of the varied levels of experience of the participating residents and that evaluation survey was the only tool they used to assess the success of the programme. However, it does demonstrate an appetite for realistic simulation training.

Our study demonstrates that it is feasible to integrate cadaveric surgery into core training curriculum. It is already accepted that trainees should have the opportunity to develop skills by simulation prior to operating on live patients. However, insufficient literature addresses the relationship between confidence and surgical skills [19]. The results presented here address this gap and demonstrate that cadaveric surgery may improve the confidence of trainees when introduced as an adjunct to conventional training. This is valuable as there is evidence that improved confidence in a task enhances self-efficacy [15]. Furthermore, an association between surgical skills and confidence has both educational and clinical implications [19]. We recognise that this is a study with a small sample size; however, using a mixed methods approach, we have assessed the complex construct of confidence and plan further studies with a larger cohort of trainees.

We acknowledge that surgical confidence depends on many factors within a training environment. We were not able to determine the relative merits of procedure-specific cadaver training versus dedicated skill training in the lab with a consultant surgeon in this study. Also, no comparison was made with other synthetic or virtual reality training tools. However, the results demonstrate that cadaveric surgical training is well received by trainees and appears to be an effective intervention.

In light of the findings of this study, we can conclude that it is feasible to integrate cadaveric surgical training into conventional gynaecology surgical training. Our next priority would be to reinforce the hypothesis of quantitative confidence improvements by incorporating a larger cohort of delegates.

This study gives some support to the notion that simulation training improves surgical technique; however, the concept of surgical competence is complex and whether simulation is an effective tool to teach or assess competence is beyond the scope of this study. It is therefore important to acknowledge that although cadaveric surgical training can serve as a valid adjunct to conventional training, it does not supersede live surgical training. This is expected to be addressed in the more senior years of training in the clinical environment.

## Conclusions

It is pragmatically feasible to provide procedure-specific cadaveric surgical training alongside supervised clinical training. Assessment of this training can be achieved by a mixed quantitative and qualitative approach. This study will form the basis of a larger study. This work has implications surrounding the improvement of self-confidence and assisting educators in implementing interventions to optimise success and self-confidence during surgical skills acquisition.
