# Correction to: Cadaveric surgery in core gynaecology training: a feasibility study

**DOI:** 10.1186/s10397-018-1038-4

**Published:** 2018-02-19

**Authors:** Chou Phay Lim, Mark Roberts, Tony Chalhoub, Jason Waugh, Laura Delgaty

**Affiliations:** 1 Obstetrics and Gynaecology, Royal Victoria Infirmary, Newcastle upon Tyne Hospitals NHS Foundation Trust, Newcastle Upon Tyne, NE1 4LP UK; 2 Royal Victoria Infirmary, Newcastle Upon Tyne, UK; 3 Obstetrics and Gynaecology, Northern Deanery, HEE (NE), Newcastle Upon Tyne, UK; 4 Newcastle University, Newcastle Upon Tyne, UK

## Correction

After publication of the original article [1] it was brought to our attention that author Laura Delgaty was incorrectly included as Laura Delegate. The correct spelling of the author’s name is included in the author list of this correction and updated in the original article.
